# (*E*)-3-(2-Chloro­phen­yl)-1-(2-fur­yl)prop-2-en-1-one

**DOI:** 10.1107/S1600536808020965

**Published:** 2008-07-12

**Authors:** Hoong-Kun Fun, P. S. Patil, Samuel Robinson Jebas, S. M. Dharmaprakash

**Affiliations:** a X-ray Crystallography Unit, School of Physics, Universiti Sains Malaysia, 11800 USM, Penang, Malaysia; b Department of Studies in Physics, Mangalore University, Mangalagangotri, Mangalore 574 199, India

## Abstract

The title compound, C_13_H_9_ClO_2_, adopts an *E* configuration with respect to the C=C double bond of the propenone unit. The benzene and furyl rings are twisted slightly from each other, making a dihedral angle of 6.47 (7)°. Intra­molecular C—H⋯O and C—H⋯Cl hydrogen bonds generate an *S*(5)*S*(5)*S*(5) ring motif. In the crystal structure, mol­ecules are stacked along the *b* axis and weak inter­molecular C—H⋯O hydrogen bonds are observed.

## Related literature

For related literature on chalcone derivatives, see: Patil *et al.* (2006 ▶); Patil, Ng *et al.* (2007 ▶); Patil, Fun *et al.* (2007 ▶). For bond-length data, see: Allen *et al.* (1987 ▶); Fun *et al.* (2008 ▶). For graph-set analysis of hydrogen bonding, see: Bernstein *et al.* (1995 ▶).
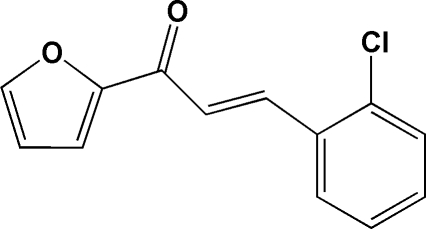


## Experimental

### 

#### Crystal data

C_13_H_9_ClO_2_
*M* *_r_* = 232.65
Orthorhombic, 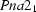

*a* = 19.6826 (4) Å
*b* = 3.8395 (1) Å
*c* = 14.0491 (3) Å
*V* = 1061.71 (4) Å^3^
*Z* = 4
Mo *K*α radiation
μ = 0.34 mm^−1^
*T* = 100.0 (1) K
0.44 × 0.23 × 0.15 mm

#### Data collection

Bruker SMART APEXII CCD area-detector diffractometer
Absorption correction: multi-scan (**SADABS**; Bruker, 2005 ▶) *T*_min_ = 0.865, *T*_max_ = 0.952
30354 measured reflections
3902 independent reflections
3738 reflections with *I* > 2σ(*I*)
*R*_int_ = 0.034

#### Refinement

*R*[*F* ^2^ > 2σ(*F* ^2^)] = 0.032
*wR*(*F* ^2^) = 0.092
*S* = 1.09
3902 reflections
145 parameters
1 restraint
H-atom parameters constrained
Δρ_max_ = 0.42 e Å^−3^
Δρ_min_ = −0.19 e Å^−3^
Absolute structure: Flack (1983 ▶), 1881 Friedel pairs
Flack parameter: −0.01 (4)

### 

Data collection: *APEX2* (Bruker, 2005 ▶); cell refinement: *APEX2*; data reduction: *SAINT* (Bruker, 2005 ▶); program(s) used to solve structure: *SHELXTL* (Sheldrick, 2008 ▶); program(s) used to refine structure: *SHELXTL*; molecular graphics: *SHELXTL*; software used to prepare material for publication: *SHELXTL* and *PLATON* (Spek, 2003 ▶).

## Supplementary Material

Crystal structure: contains datablocks global, I. DOI: 10.1107/S1600536808020965/is2312sup1.cif

Structure factors: contains datablocks I. DOI: 10.1107/S1600536808020965/is2312Isup2.hkl

Additional supplementary materials:  crystallographic information; 3D view; checkCIF report

## Figures and Tables

**Table 1:** Hydrogen-bond geometry (Å, °)

*D*—H⋯*A*	*D*—H	H⋯*A*	*D*⋯*A*	*D*—H⋯*A*
C3—H3*A*⋯O2^i^	0.93	2.52	3.4126 (14)	161
C11—H11*A*⋯O2^ii^	0.93	2.55	3.1488 (17)	123
C7—H7*A*⋯Cl1	0.93	2.64	3.0675 (12)	108
C7—H7*A*⋯O2	0.93	2.50	2.8255 (14)	101
C8—H8*A*⋯O1	0.93	2.49	2.8249 (15)	101

Symmetry codes: (i) 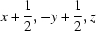; (ii) 
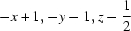
.

**Fig. 1.**
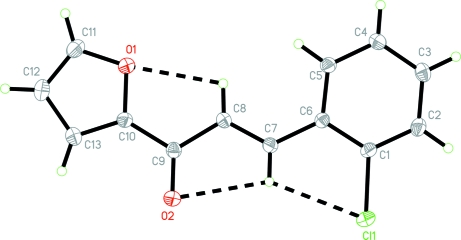
The molecular structure of the title compound, showing 50% probability displacement ellipsoids and the atom numbering scheme. Hydrogen bonds are shown as dashed lines.

**Fig. 2.**
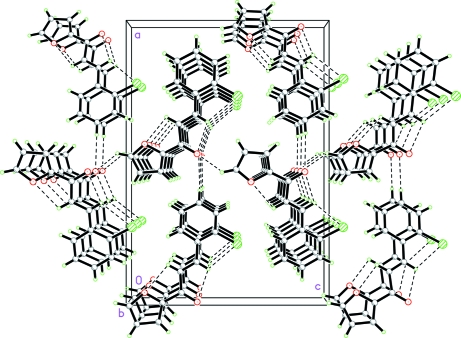
The crystal packing of the title compound, viewed along the b axis. Hydrogen bonds are shown as dashed lines.

## supplementary crystallographic information

### Comment

The title compound, (I), whose structure is reported here, is a chalcone derivative that we have prepared; the crystal structures of some of these compounds have been studied previously (Patil *et al.*, 2006; Patil, Ng *et al.*, 2007; Patil, Fun *et al.*, 2007).

In (I), the moleule exhibits an *E* configuration with respect to the C7═C8 double bond with the C6—C7—C8—C9 torsion angle being 178.45 (10)°. The bond lengths and bond angles in (I) are found to have normal values (Allen *et al.*, 1987; Fun *et al.*, 2008). The phenyl and furyl rings in the molecule is planar with the maximum deviation from planarity being 0.005 (13) Å for atom C6 and 0.004 (11) Å for atom O1, respectively. The dihedral angle between the phenyl and the furyl ring are 6.47 (7)°, indicating that they are slightly twisted from each other. The non-centrosymmmetric crystal of the title compound should exhibit 2nd-order NLO properties.

Intramolecular C—H···O and C—H···Cl hydrogen bonds generate an S(5)S(5)S(5) ring motif (Bernstein *et al.*, 1995). In the crystal structure, the molecules are stacked along the *b* axis. The crystal packing is consolidated by inter and intramolecular C—H···O and C—H···Cl hydrogen bonding interactions.

### Experimental

The compound (I) was synthesized by the condensation of 2-chlorobenzaldehyde (0.01 mol, 1.49 mg) with 2-acetylfuran (0.01 mol, 1.01 ml) in methanol (60 ml) in the presence of a catalytic amount of sodium hydroxide solution (5 ml, 30%). After stirring (6 h), the contents of the flask were poured into ice-cold water (500 ml) and left to stand for 5 h. The resulting crude solid was filtered and dried. The precipitated compound was recrystallized from N, *N*-dimethylformamide (DMF).

### Refinement

H atoms were positioned geometrically (C—H = 0.93 Å) and refined using a riding model, with *U*_iso_(H) = 1.2*U*_eq_(C).

### Crystal data

**Table:**

C_13_H_9_ClO_2_	*F*_000_ = 480
*M**_r_* = 232.65	*D*_x_ = 1.455 Mg m^−^^3^
Orthorhombic, *P**n**a*2_1_	Mo *K*α radiation λ = 0.71073 Å
Hall symbol: P 2c -2n	Cell parameters from 9970 reflections
*a* = 19.6826 (4) Å	θ = 2.5–37.2º
*b* = 3.8395 (1) Å	µ = 0.34 mm^−^^1^
*c* = 14.0491 (3) Å	*T* = 100.0 (1) K
*V* = 1061.71 (4) Å^3^	Block, colourless
*Z* = 4	0.44 × 0.23 × 0.15 mm

### Data collection

**Table:**

Bruker SMART APEXII CCD area-detector diffractometer	3902 independent reflections
Radiation source: fine-focus sealed tube	3738 reflections with *I* > 2σ(*I*)
Monochromator: graphite	*R*_int_ = 0.034
*T* = 100.0(1) K	θ_max_ = 32.8º
φ and ω scans	θ_min_ = 2.1º
Absorption correction: multi-scan(*SADABS*; Bruker, 2005)	*h* = −29→29
*T*_min_ = 0.865, *T*_max_ = 0.952	*k* = −5→5
30354 measured reflections	*l* = −21→21

### Refinement

**Table:**

Refinement on *F*^2^	Hydrogen site location: inferred from neighbouring sites
Least-squares matrix: full	H-atom parameters constrained
*R*[*F*^2^ > 2σ(*F*^2^)] = 0.032	*w* = 1/[σ^2^(*F*_o_^2^) + (0.06*P*)^2^ + 0.0834*P*] where *P* = (*F*_o_^2^ + 2*F*_c_^2^)/3
*wR*(*F*^2^) = 0.092	(Δ/σ)_max_ = 0.001
*S* = 1.10	Δρ_max_ = 0.42 e Å^−^^3^
3902 reflections	Δρ_min_ = −0.19 e Å^−^^3^
145 parameters	Extinction correction: none
1 restraint	Absolute structure: Flack (1983), 1881 Friedel pairs
Primary atom site location: structure-invariant direct methods	Flack parameter: −0.01 (4)
Secondary atom site location: difference Fourier map	

### Special details

**Table:**

Experimental. The data was collected with the Oxford Cyrosystem Cobra low-temperature attachment.
Geometry. All e.s.d.'s (except the e.s.d. in the dihedral angle between two l.s. planes) are estimated using the full covariance matrix. The cell e.s.d.'s are taken into account individually in the estimation of e.s.d.'s in distances, angles and torsion angles; correlations between e.s.d.'s in cell parameters are only used when they are defined by crystal symmetry. An approximate (isotropic) treatment of cell e.s.d.'s is used for estimating e.s.d.'s involving l.s. planes.
Refinement. Refinement of *F*^2^ against ALL reflections. The weighted *R*-factor *wR* and goodness of fit *S* are based on *F*^2^, conventional *R*-factors *R* are based on *F*, with *F* set to zero for negative *F*^2^. The threshold expression of *F*^2^ > σ(*F*^2^) is used only for calculating *R*-factors(gt) *etc*. and is not relevant to the choice of reflections for refinement. *R*-factors based on *F*^2^ are statistically about twice as large as those based on *F*, and *R*- factors based on ALL data will be even larger.

### Fractional atomic coordinates and isotropic or equivalent isotropic displacement parameters (Å^2^)

**Table:**

	*x*	*y*	*z*	*U*_iso_*/*U*_eq_	
Cl1	0.717808 (15)	0.28404 (8)	0.54723 (2)	0.02583 (8)	
O1	0.56257 (5)	−0.2638 (2)	0.10796 (7)	0.02292 (18)	
O2	0.52989 (4)	−0.2045 (3)	0.35622 (8)	0.02328 (19)	
C13	0.47093 (6)	−0.4899 (3)	0.17874 (10)	0.0209 (2)	
H13A	0.4398	−0.5572	0.2251	0.025*	
C1	0.75871 (6)	0.3597 (3)	0.43900 (8)	0.01692 (19)	
C2	0.82334 (6)	0.5030 (3)	0.44283 (9)	0.0203 (2)	
H2A	0.8429	0.5581	0.5012	0.024*	
C3	0.85871 (6)	0.5636 (3)	0.35885 (10)	0.0205 (2)	
H3A	0.9022	0.6575	0.3606	0.025*	
C4	0.82839 (6)	0.4826 (3)	0.27213 (9)	0.0188 (2)	
H4A	0.8516	0.5252	0.2157	0.023*	
C5	0.76378 (6)	0.3389 (3)	0.26935 (8)	0.0173 (2)	
H5A	0.7444	0.2862	0.2107	0.021*	
C6	0.72688 (6)	0.2709 (3)	0.35301 (9)	0.01496 (19)	
C7	0.65913 (5)	0.1151 (3)	0.35030 (8)	0.01663 (18)	
H7A	0.6348	0.1105	0.4070	0.020*	
C8	0.62901 (5)	−0.0215 (3)	0.27343 (8)	0.0177 (2)	
H8A	0.6512	−0.0168	0.2150	0.021*	
C9	0.56065 (6)	−0.1798 (3)	0.28067 (8)	0.01660 (19)	
C10	0.53018 (6)	−0.3151 (3)	0.19325 (9)	0.0167 (2)	
C11	0.52194 (7)	−0.4069 (4)	0.03974 (10)	0.0256 (2)	
H11A	0.5316	−0.4062	−0.0251	0.031*	
C12	0.46571 (7)	−0.5500 (4)	0.07910 (10)	0.0238 (2)	
H12A	0.4307	−0.6644	0.0474	0.029*	

### Atomic displacement parameters (Å^2^)

**Table:**

	*U*^11^	*U*^22^	*U*^33^	*U*^12^	*U*^13^	*U*^23^
Cl1	0.02834 (15)	0.03521 (16)	0.01394 (11)	−0.00595 (11)	0.00082 (12)	0.00028 (14)
O1	0.0203 (4)	0.0318 (5)	0.0166 (4)	−0.0043 (3)	0.0011 (3)	−0.0022 (3)
O2	0.0198 (4)	0.0327 (5)	0.0174 (4)	−0.0050 (3)	0.0022 (3)	−0.0006 (4)
C13	0.0175 (5)	0.0221 (5)	0.0231 (5)	−0.0020 (4)	−0.0007 (4)	−0.0002 (4)
C1	0.0184 (5)	0.0190 (4)	0.0134 (4)	0.0011 (4)	−0.0005 (4)	0.0002 (4)
C2	0.0191 (5)	0.0228 (5)	0.0189 (5)	−0.0005 (4)	−0.0045 (4)	−0.0002 (4)
C3	0.0152 (4)	0.0215 (5)	0.0249 (5)	−0.0003 (4)	−0.0026 (4)	0.0012 (4)
C4	0.0169 (5)	0.0198 (5)	0.0196 (5)	0.0003 (4)	0.0013 (4)	0.0026 (4)
C5	0.0168 (5)	0.0191 (5)	0.0159 (5)	0.0005 (4)	−0.0001 (4)	0.0005 (4)
C6	0.0146 (4)	0.0153 (4)	0.0149 (5)	0.0013 (3)	−0.0011 (4)	0.0004 (4)
C7	0.0156 (4)	0.0183 (4)	0.0160 (4)	−0.0008 (3)	0.0001 (4)	0.0013 (4)
C8	0.0145 (4)	0.0211 (5)	0.0177 (5)	−0.0015 (4)	0.0005 (4)	−0.0010 (4)
C9	0.0151 (4)	0.0173 (4)	0.0174 (5)	0.0005 (3)	−0.0006 (4)	0.0000 (4)
C10	0.0162 (5)	0.0180 (5)	0.0160 (5)	0.0003 (3)	0.0010 (4)	−0.0005 (4)
C11	0.0267 (6)	0.0319 (6)	0.0182 (5)	−0.0006 (5)	−0.0020 (5)	−0.0040 (5)
C12	0.0221 (5)	0.0231 (5)	0.0263 (6)	−0.0013 (4)	−0.0063 (4)	−0.0036 (5)

### Geometric parameters (Å, °)

**Table:**

Cl1—C1	1.7449 (12)	C4—C5	1.3868 (16)
O1—C11	1.3637 (16)	C4—H4A	0.9300
O1—C10	1.3715 (15)	C5—C6	1.4061 (17)
O2—C9	1.2256 (15)	C5—H5A	0.9300
C13—C10	1.3610 (16)	C6—C7	1.4622 (16)
C13—C12	1.4224 (19)	C7—C8	1.3389 (16)
C13—H13A	0.9300	C7—H7A	0.9300
C1—C2	1.3869 (17)	C8—C9	1.4800 (15)
C1—C6	1.4030 (16)	C8—H8A	0.9300
C2—C3	1.3895 (18)	C9—C10	1.4621 (16)
C2—H2A	0.9300	C11—C12	1.3537 (19)
C3—C4	1.3918 (18)	C11—H11A	0.9300
C3—H3A	0.9300	C12—H12A	0.9300
			
C11—O1—C10	106.48 (10)	C1—C6—C7	121.95 (11)
C10—C13—C12	106.81 (11)	C5—C6—C7	121.69 (11)
C10—C13—H13A	126.6	C8—C7—C6	125.81 (11)
C12—C13—H13A	126.6	C8—C7—H7A	117.1
C2—C1—C6	122.63 (11)	C6—C7—H7A	117.1
C2—C1—Cl1	117.10 (9)	C7—C8—C9	120.53 (11)
C6—C1—Cl1	120.26 (9)	C7—C8—H8A	119.7
C1—C2—C3	119.54 (11)	C9—C8—H8A	119.7
C1—C2—H2A	120.2	O2—C9—C10	119.82 (10)
C3—C2—H2A	120.2	O2—C9—C8	122.72 (11)
C2—C3—C4	119.41 (10)	C10—C9—C8	117.46 (10)
C2—C3—H3A	120.3	C13—C10—O1	109.77 (11)
C4—C3—H3A	120.3	C13—C10—C9	130.74 (11)
C5—C4—C3	120.44 (11)	O1—C10—C9	119.49 (10)
C5—C4—H4A	119.8	C12—C11—O1	110.84 (12)
C3—C4—H4A	119.8	C12—C11—H11A	124.6
C4—C5—C6	121.60 (11)	O1—C11—H11A	124.6
C4—C5—H5A	119.2	C11—C12—C13	106.10 (11)
C6—C5—H5A	119.2	C11—C12—H12A	127.0
C1—C6—C5	116.36 (10)	C13—C12—H12A	127.0
			
C6—C1—C2—C3	0.14 (18)	C7—C8—C9—O2	−2.60 (17)
Cl1—C1—C2—C3	178.98 (9)	C7—C8—C9—C10	178.35 (11)
C1—C2—C3—C4	0.63 (17)	C12—C13—C10—O1	−0.30 (14)
C2—C3—C4—C5	−0.74 (18)	C12—C13—C10—C9	178.44 (12)
C3—C4—C5—C6	0.08 (18)	C11—O1—C10—C13	0.67 (14)
C2—C1—C6—C5	−0.76 (16)	C11—O1—C10—C9	−178.23 (11)
Cl1—C1—C6—C5	−179.57 (9)	O2—C9—C10—C13	−3.41 (19)
C2—C1—C6—C7	179.05 (11)	C8—C9—C10—C13	175.66 (12)
Cl1—C1—C6—C7	0.24 (15)	O2—C9—C10—O1	175.22 (11)
C4—C5—C6—C1	0.65 (16)	C8—C9—C10—O1	−5.70 (15)
C4—C5—C6—C7	−179.17 (10)	C10—O1—C11—C12	−0.80 (15)
C1—C6—C7—C8	−170.09 (11)	O1—C11—C12—C13	0.62 (15)
C5—C6—C7—C8	9.72 (17)	C10—C13—C12—C11	−0.19 (15)
C6—C7—C8—C9	178.45 (10)		

### Hydrogen-bond geometry (Å, °)

**Table:**

*D*—H···*A*	*D*—H	H···*A*	*D*···*A*	*D*—H···*A*
C3—H3A···O2^i^	0.93	2.52	3.4126 (14)	161
C11—H11A···O2^ii^	0.93	2.55	3.1488 (17)	123
C7—H7A···Cl1	0.93	2.64	3.0675 (12)	108
C7—H7A···O2	0.93	2.50	2.8255 (14)	101
C8—H8A···O1	0.93	2.49	2.8249 (15)	101

Symmetry codes: (i) *x*+1/2, −*y*+1/2, *z*; (ii) −*x*+1, −*y*−1, *z*−1/2.
